# Green Synthesis of Zinc Oxide Nanoparticles from Pomegranate (*Punica granatum*) Extracts and Characterization of Their Antibacterial Activity

**DOI:** 10.3390/molecules25194521

**Published:** 2020-10-02

**Authors:** Ugochi Lydia Ifeanyichukwu, Omolola Esther Fayemi, Collins Njie Ateba

**Affiliations:** 1 Food Security and Safety Niche Area, Faculty of Natural and Agricultural Sciences, North West University, Private Bag X2046, Mmabatho 2735, South Africa; lydiaifeanyichukwu@gmail.com; 2 Department of Chemistry, Faculty of Natural and Agricultural Sciences, North West University, Private Bag X2046, Mmabatho 2735, South Africa; Omolola.Fayemi@nwu.ac.za

**Keywords:** biosynthesis, nanoparticles, biocontrol, antimicrobial efficacy

## Abstract

This study assessed the antimicrobial efficacy of synthesized zinc oxide nanoparticles produced using aqueous extracts of pomegranate leaves and flowers designated ZnO-NPs-PL, ZnO-NPs-PF. In the study, oxides of zinc were successfully employed to fabricate nanoparticles using extracts from leaves and flowers of pomegranate (*Punica granatum*). The nanoparticles obtained were characterized spectroscopically. X-ray diffractive analysis (XRD) revealed the elemental components and nature of the synthesized particles. The fabricated zinc oxide nanoparticle (ZnO-NPs) showed a crystalline structure. The morphology of the nanoparticles as shown by scanning electron microscopy (SEM) was unevenly spherical and the functional groups involved in stabilization, reduction and capping were confirmed using Fourier Transform Infra-Red (FT-IR) Spectroscopy. Confirmation of the nanoparticles by UV–Vis analysis showed absorption bands of 284 and 357 nm for pomegranate leaf and flower extract, respectively, mediated ZnO-NPs. Evaluation of the antimicrobial efficacy of the fabricated nanoparticles showed that ZnO-NPs were effective against all selected pathogenic strains, *Staphylococcus aureus*, *Bacillus cereus*, *Pseudomonas aeruginosa*, *Klebsiella pneumoniae*, *Streptococcus pneumoniae*, *Salmonella diarizonae*, *Salmonella typhi*, *Enterococcus faecalis*, *Enterococcus faecium*, *Escherichia coli*, *Moraxella catarrhalis*, *Aeromonas hydrophila and Listeria monocytogenes*, used in the analysis. The effectiveness of these nanoparticles could be linked to their sizes and shapes as obtained using a transmission electron microscope (TEM) and scanning electron microscope (SEM). Our reports revealed that increasing the concentration of the nanoparticles resulted in an increase in the antibacterial activity exerted by the nanoparticles, thus suggesting that both ZnO-NPs can effectively be used as alternative antibacterial agents. Further research is required to assess their mechanisms of action and toxicity.

## 1. Introduction

Bacterial pathogens cause infections that are typically treated using antibiotics [1,2]. Antibiotics are antibacterial agents that inhibit the growth of bacterial pathogens through diffident modes of action ranging from inhibition of enzyme action to interfering with DNA, RNA and protein synthesis [3]. These processes ultimately result in the disruption of the bacterial cell membrane structure, leading to cell death [2,3]. Recent studies have demonstrated that bacterial pathogens are exhibiting resistance against a variety of antibiotics [4], thereby limiting the effectiveness of these agents [5]. Contrary to the modes of action of antibiotics, bacterial strains are able to express antimicrobial resistance by: (1) altering the target of antibiotics by expressing genes that code for an alternate version of the antibiotic target [6,7,8]; (2) developing enzymes that can degrade or modify the drug [1,8]; (3) ensuring reduced uptake of antimicrobial drugs or acting as efflux pumps that push out the drugs [3,6]; and (4) formation of biofilm layers around the bacterial cell, thus limiting or reducing its exposure to antibiotics [8,9]. The ever-increasing occurrence of bacterial resistance among pathogenic bacteria that is most often caused by the inappropriate or misuse of antibiotics [10], coupled with limited surveillance data as well as the recent increase in biofilm-associated infections in humans, has led to the search for more effective agents and strategies to combat antimicrobial resistance [11,12].

Owing to their antibacterial activity, different inorganic metals and their oxides, such as zinc, zinc oxide, copper, copper oxide, titanium, titanium oxide, magnesium and magnesium oxide, are being exploited as potential antimicrobial agents since they are stable, robust and have a long shelf life [10,13,14]. Metals and metal oxides have been used in the past for treatment of infections and illnesses [9,15,16]. Metals exert their antimicrobial strength on Gram-positive and Gram-negative bacteria by selectively upsetting the process required for cell growth [1,17]. The antimicrobial mechanism of action of metal oxides involves the release of metal ions, which are absorbed by microbial cell membranes, eliciting interactions with functional groups of protein and nucleic acid, thereby inhibiting enzyme activity [2,9]. This results in a change in cell structure and finally inhibits the microorganism [2,9]. In addition to these, another mechanism of action exhibited by metal oxides is the production of reactive oxygen species (ROS), which triggers electrostatic interaction, thus altering the prokaryotic cell wall and enzyme or DNA pathways [1,8].

Nanoparticles are small objects with a diameter of about 1–100 nm and they have a wide range of applications [2,14]. There is a possibility to achieve metal and metal oxide nanoparticles [18] which have applications in medicine, biotechnology and many other industrial applications [19]. In recent times, formulated metal and metal oxide nanoparticles, especially those synthesized from plant extracts, have been explored as alternative bio-control agents [3,5,10,20]. In addition, these biosynthesized metal and metal oxide nanoparticles have produced very valuable results when utilized as treatment agents against some cases of infections caused by multidrug-resistant Gram-negative and Gram-positive bacteria [21,22]. In the biosynthesis of nanoparticles, the plant extracts function as reducing, stabilizing and capping agents [8,23,24].

The pomegranate plant is reported to possess important biomolecules and metabolites, including organic acids, polyphenols, flavonoids, anthocyanins, alkaloids, fatty acids and vitamins [19]. The high phenolic content of pomegranate has been reported to account for its antimicrobial, anthelmintic, anti-inflammatory and antioxidant properties [25]. However, the effectiveness of synthesized metal and metal oxide nanoparticles is greatly related to their stability, size, size distribution, surface functionality, morphology, shape and the type of material used in synthesis [12,26,27,28].

As stated earlier, studies have shown that the smaller the size of the nanoparticle, the larger its surface to volume ratio, and thus the higher its antimicrobial efficacy [2,5,18,22]. This is because small nanoparticles can interact directly with the cell membrane of bacterial pathogens [29]. In terms of shape, sphere-shaped nanoparticles have been shown to have more antibacterial capabilities [30,31]. Nanoparticles can interact with biological molecules; this can give them biomedical applications, especially in the area of cancer diagnosis and treatment [32].

The zinc oxide nanoparticle is one of the most studied inorganic metal oxide nanoparticles [28], and this is attributed to its stability under harsh conditions, predominant antimicrobial properties and low toxicity to humans [22,28,33]. Several studies have shown that zinc oxide nanoparticles have great antimicrobial activity against several microorganisms [4] and may prevent biofilm formation [23]. In a previous study, Akbar et al. [33] synthesized 20 nm-sized zinc oxide nanoparticles that were tested against *Salmonella typhimurium* and *Staphylococcus aureus*, and reported that the nanoparticles showed potent antimicrobial effects against the tested bacteria. Salem et al. [14] also evaluated the antimicrobial effects of zinc oxide nanoparticles against *Vibrio cholera* and enterotoxic *Escherichia coli*, while Chaudhary et al. [34] assessed the antimicrobial effect of zinc oxide nanoparticles against the following pathogenic organisms: *Staphylococcus epidermidis* (MTCC-3382), *Staphylococcus epidermidis* (MTCC-3382), *Klebsiella pneumoniae* (MTCC-3384), *Escherichia coli* (MTCC-41) and fungi, *Aspergillus niger* (MTCC-404) and *Aspergillus oryzae* (MTCC-3107).

## 2. Materials and Methods

Zinc nitrate hexahydrate [Zn (NO_3_)_2_·6H_2_O] (Merck, Kenilworth, NJ, USA) was used as a precursor to synthesize zinc oxide nanoparticles. Tryptic soy broth and Mueller–Hinton agar (Sigma-Aldrich, St. Louis, MO, USA). All solutions were prepared using sterile distilled water. The bacterial strains used in this study were American Type Culture Collection strains purchased from BioMérieux South Africa while the *Salmonella typhi* were obtained from the Microbiology Culture Collection Unit at the Department of Microbiology, North-West University, South Africa. The bacteria species, strain numbers and the morphological characteristics are outlined in Table 1 below.

### 2.1. Collection and Preparation of Pomegranate Leaf and Flower Extract

**Figure 1:**
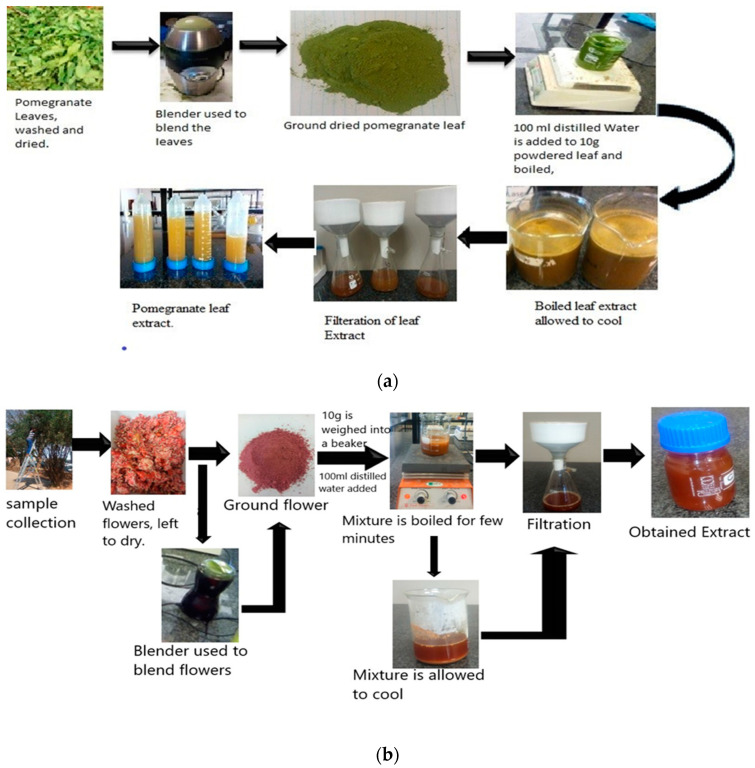
(**a**) Pictorial representation of the preparation process for pomegranate (*Punica granatum*) leaf extract. (**b**) Pictorial representation of the preparation process for pomegranate (*Punica granatum*) flower extract.

Healthy leaves and flowers of pomegranates were collected from Unit 6 in Mafikeng, North-West Province, South Africa. The leaves and flowers were washed separately with tap water to remove dust particles, then washed again with distilled water and dried for 4–6 days. The dried leaves and flowers were each separately shredded and ground to a fine powder, then stored in a properly labelled bottle for further use. Ten grams of the leaf and flower powder were weighed out and put into a well-labelled beaker, and 100 mL sterile double distilled water were added to each. The flower and leaf mixtures were heated for 20 min at 60 °C. The obtained extract was allowed to cool down and filtered using Whatman filter paper. The filtrate was collected in a well-labeled Erlenmeyer flask and stored at 4 °C for further use. Figure 1 represents pictorial representation of the extract production process.

### 2.2. Synthesis of Zinc Oxide Nanoparticles

**Figure 2:**
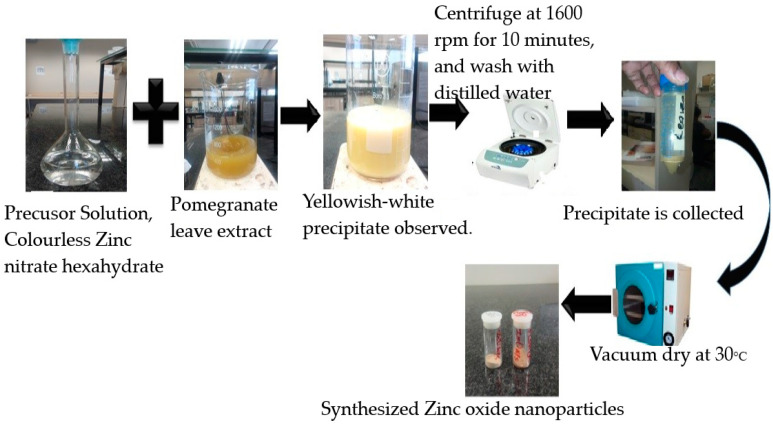
Synthesis of zinc oxide nanoparticles via pomegranate leaf extracts (pictorial representation).

The 0.1 M zinc nitrate hexahydrate (Zn (NO_3_)_2_·6H_2_O) solution was prepared by dissolving 6.58 g in 300 mL double-distilled water. Ten milliliters of the aqueous leaf and flower pomegranate extracts were each slowly added dropwise into the solution under magnetic stirring at 60 °C for roughly 2 h to obtain complex formation. The complex formed after stirring was collected and centrifuged at 10,000 rpm for 10 min and the pellets were collected as shown in Figure 2. The separated pellets were dried in an oven at 80 °C for 8 h and preserved in airtight bottles for further studies.

Biosynthesis of zinc oxide nanoparticles was carried out using pomegranate leaves and flower extract and zinc nitrate hexahydrate as a precursor. On addition of the plant extracts (leaf and flower) with a greenish and pinkish-brown color, respectively, to the colorless zinc nitrate hexahydrate solution, a yellowish-white precipitate occurred, indicating the presence of zinc oxide nanoparticles. A similar color change was observed by Rajakumar et al. [35] on addition of greenish *Andrographis paniculata* leaf extract to the zinc nitrate precursor. Bhuyan et al. [36] also reported formation of a whitish precipitate. In contrast, Umar et al. [37] observed a change in color from brown to a dark brown color on addition of *Albizia lebbeck* leaf extract to the precursor zinc acetate.

### 2.3. Characterization of the Synthesized Metal Oxide Nanoparticles

The resulting synthesized metal oxide nanoparticles were characterized by UV–visible spectroscopy, X-ray diffraction (XRD), scanning electron microscopy (SEM), transmission electron microscopy (TEM) and Fourier transform infrared spectrophotometry (FTIR). UV–Vis analysis was conducted using a UV–visible spectrometer (Aglient Technologies Inc., Santa Clara, CA, USA), to determine the formation and stability of nanoparticles. The zinc oxide nanoparticles synthesized from pomegranate leaves and flowers were weighed out (2 µg) and distilled water was added, then sonicated. The resulting solutions were poured into cuvettes and spectra in the range of 200–800 nm were determined. The X-ray diffraction analysis was done to obtain information about the morphology and crystalline nature of the formed particles. XRD measures symmetry, size and shape. Synthesized nanoparticles were analyzed in powdered form using an X-ray diffractometer (Brucker, Billerica, MA, USA) of angle 2Ɵ in the range 20–80°. The Cary 600 series FTIR spectrometer (Opus Alpha-P, Brucker Corporation, Billerica, MA, USA) was used to identify the FTIR spectra of the powdered samples of the synthesized metal oxide nanoparticles. Additionally, FTIR analysis was carried out so as to detect the surface functional groups and the stretching and bending vibrations in the pomegranate extract and the nanomaterial samples. The morphology, microstructure and the elemental composition of the synthesized metal oxide nanoparticles was done using scanning electron microscopy (SEM) analysis with energy dispersive X-ray analysis (EDX). The SEM images were recorded using a Hitachi 3600 SEM (Shizuoka, Japan) instrument and energy dispersive X-ray analysis (EDX) was done by using the Thermo Fisher Scientific Ultra-dry (Madison, WI, USA) instrument. TEM analysis was done to provide information on the shape and size distribution of synthesized nanoparticles while confirming the existence of metal oxide nanoparticles in the synthesized samples. A JEOLJEM 2100 electron microscope (JOEL ltd, TYO, Japan) operated at 200kv accelerating voltage and connected to an energy dispersive spectrophotometer (EDX) was used for TEM analysis. In performing the analysis, 2 mg of each sample were weighed out into a sample bottle and about 4 mL ethanol solution were added. The mixture was sonicated for 10 min to disperse the particles, using a digital ultrasonic cleaner. A drop of the sonicated mixture was placed on a carbon-coated copper grid and allowed to dry for about 5 min.

### 2.4. Preparation of Nanoparticle Suspensions

Varying concentrations of synthesized metal oxide nanoparticles (50, 100, 1000 and 5000 µg/mL) were prepared by adding a known quantity (µg or mg) of each particle (ZnO-NPs-Pomegranate Leaf (ZnO-NPs-PL), ZnO-NPs-Pomegranate Flower (ZnO-NPs-PF)) to a measured volume of dimethyl sulfoxide (DMSO) organic solvent. The mixture was allowed to sonicate for a few minutes.

### 2.5. Antimicrobial Activity of the Synthesized Metal Oxide Nanoparticles

The antimicrobial activities of the synthesized zinc oxide nanoparticles were evaluated against thirteen pathogenic bacterial strains that are frequently associated with both food poisoning diseases and nosocomial infections in humans.

### 2.6. Preparation of Bacterial Inocula

Each pure bacterial strain was sub-cultured in tryptic soy broth (TSB) and incubated aerobically at 37 °C for 24 h. The absorbance of the bacterial cultures was adjusted to attain a viable cell count of 10^7^ CFU/mL (optical density of 0.4–0.5) at 630 nm using a spectrophotometer (model, MB-580, Shenzhen Huisong Technology Development Co., Ltd., Shenzhen, China).

### 2.7. Antibiogram Test of Bacterial Strains

As an internal control, the bacterial strains assessed in this study were screened against standard antibiotics using the Kirby–Bauer method on Mueller–Hinton agar plates. Antibiotic discs used were: Augmentin (30 µg), gentamicin (30 µg), amikacin (30 µg), norfloxacin (10 µg), ciprofloxacin (5 µg), cefotaxime (30 µg), tetracycline (30 µg) and ampicillin (10 µg). Pre-adjusted overnight cultures (10^6^ cfu/mL) were spread-plated on Mueller–Hinton agar plates. The different antibiotic discs were placed at equitable distances on the inoculated agar using sterile forceps. The plates were incubated aerobically at 37 °C for 24 h. Antibiotic inhibition zone diameter (AIZD) data were measured in millimeters and recorded. The AIZD data were used to classify isolates as either resistant, intermediate resistant or susceptible to a particular antibiotic using standard reference values [38,39].

### 2.8. Antibacterial Activity of Biosynthesized Zinc Oxide Nanoparticles

The agar well diffusion method, as previously described by Azam et al. [40], was used to evaluate antimicrobial activities of the synthesized metal oxide nanoparticles, but with slight modifications. Aliquots of 100 µL of the 10^6^ cfu/mL bacterial culture were spread-plated on Mueller–Hinton agar and plates to prepare a lawn. The plates were left to stand for 10 min and 8 mm wells were punched on the agar. An aliquot of 100 μL of a 50 μg/mL nanoparticle solution was poured into each of well on all plates. The plates were left to stand for 1 h in the biosafety cabinet to ensure even diffusion of the samples into the agar, and later incubated at 37 °C for 24 h. Bacteria growth inhibition zone diameter data were measured in millimeters and recorded. The experiment was performed in triplicate and pooled data were statistically analyzed using the Statistical Package for the Social Sciences (SPSS), software version 20.0, IBM cooperation. Positive control, gentamicin antibiotic (30 µg) and negative control dimethyl sulfoxide were included in this experiment.

### 2.9. Determination of Minimum Inhibitory Concentration (MIC) of the Synthesized Zinc Oxide Nanoparticles

The lowest concentration of an antimicrobial agent that can inhibit microbial growth after 24 h of incubation is known as the minimum inhibitory concentration (MIC) [14]. In order to evaluate the efficiency of the synthesized nanoparticles in controlling the pathogenic bacterial growth, the most effective synthesized metal oxide nanoparticles that exhibited strong antibacterial activity at 5000 µg/mL were used to determine the MIC based on the micro-broth dilution method in 96-well microtiter plates. A two-fold dilution of the initial nanoparticle concentration (5000 µg/mL) was aseptically prepared by transferring 100 µL of the sterile nanoparticles into 100 µL of sterile tryptic soy broth in a microtiter well plate to obtain a 2500 µg/mL concentration. The process was repeated several times to obtain other concentrations (1250 µg/mL), a 625-fold dilution of the initial nanoparticle concentration (5000 µg/mL) was aseptically prepared by transferring 100 µL of the 10^6^ cfu/mL standardized bacterial suspension (OD_630 nm_ = 0.1) to the wells and the plates, which were incubated at 37 °C for 24 h. Following the overnight incubation, the optical density (OD_630_) of the plates was measured for absorbance using the HEALES full automatic microplate reader MB-580 (Shenzhen Huisong Technology), to determine the minimum inhibitory concentration that prevented growth of bacterial cells.

### 2.10. Time–Kill Kinetics Assay

The time–kill kinetics assay, also termed the antimicrobial efficacy test, is used to study the activity of an antimicrobial agent against a bacterial strain and it is used to determine the bactericidal or bacteriostatic activity of an agent over time. In order to determine the time, fabricated zinc oxide nanoparticles were reconstituted in dimethyl sulfoxide to obtain different concentrations (5 mg/mL, 2.5 mg/mL and 1.25 mg/mL). The different concentrations of the nanoparticles were transferred into the wells of a microtiter plate containing 100 µL of tryptic soy broth. An aliquot of 100 µL of the bacteria culture was added to the wells and incubated at 37 °C. Optical density was determined after every hour and recorded. The killing time kinetics that indicate the reaction of the cells when exposed to the nanoparticles were evaluated by a plot of the log of the optical density versus time.

### 2.11. Statistical Analysis

Data obtained from antimicrobial activity experiments were analyzed by one-way analysis of variance (ANOVA) using the Statistical Package for the Social Sciences (SPSS), software version 20.0, IBM cooperation, North Castle, NY, USA. Significant mean difference was compared using Duncan’s new multiple range test (DMRT) and a significant difference was defined as *p* ≤ 0.05.

## 3. Results

### 3.1. Transmission Electron Microscopy

**Figure 3:**
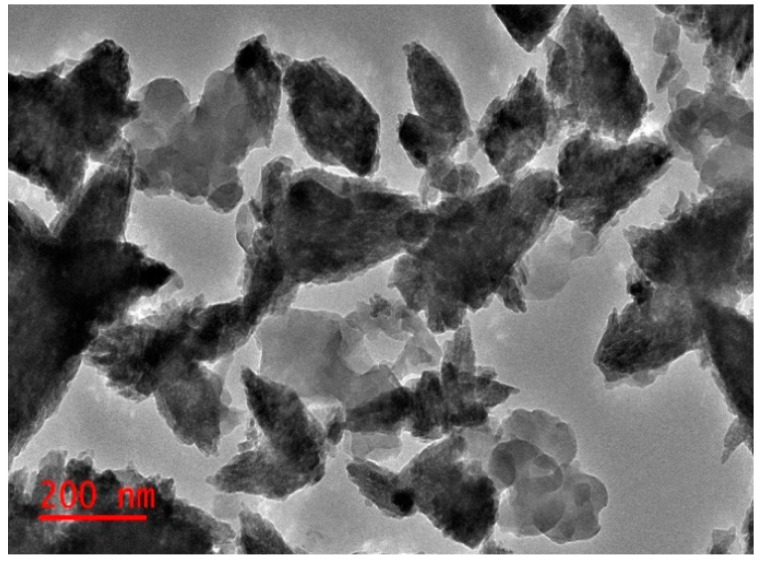
Transmission Electron Microscopy (TEM) image of synthesized ZnO-NPs-PL.

**Figure 4:**
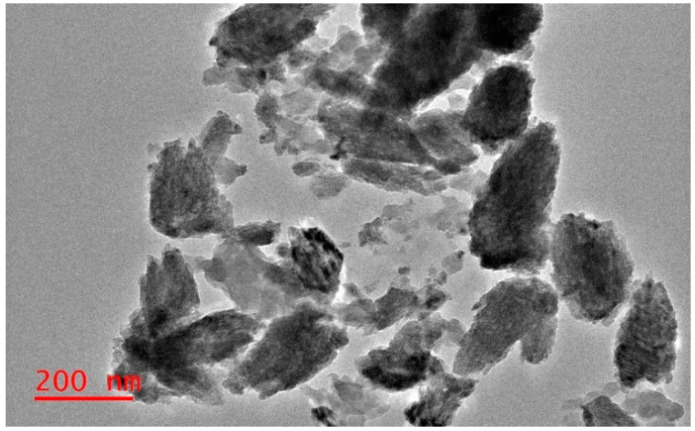
Typical TEM image of synthesized ZnO-NPs-PF.

The obtained TEM micrographs of nanoparticles showed that ZnO-NPs-PL and ZnO-NPs-PF were flower-like structures, as seen in Figure 3 and Figure 4. This conforms to the SEM images obtained for ZnO-NPs-PF. Obtained TEM images also indicated agglomeration of the particles to each other. This occurrence can be linked to the presence of biological components of the plant extract wound around the nanoparticles, resulting in particles sticking together. The TEM images showed the polydispersity of nanoparticles with spikes on the surface [41,42].

### 3.2. Ultraviolet–Visible (UV–Vis) Spectroscopy

**Figure 5:**
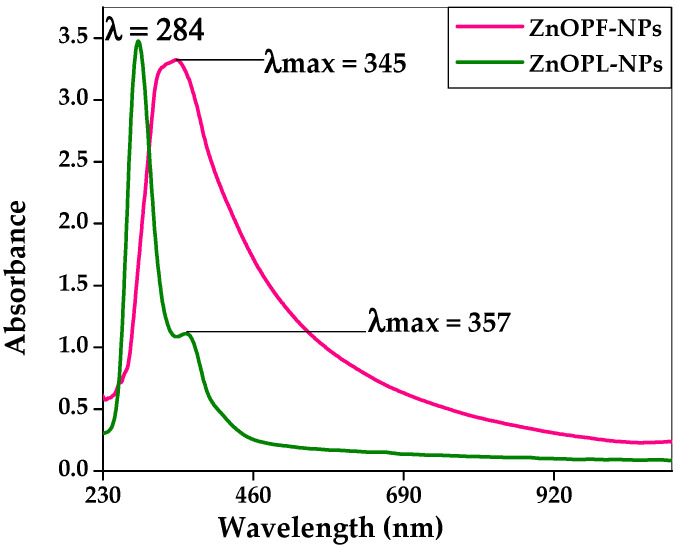
UV–visible spectra of zinc oxide nanoparticles synthesized from pomegranate leaf (ZnO-NPs-PL) and pomegranate flower (ZnO-NPs-PF) extracts.

The confirmation of formation of the zinc oxide nanoparticles was performed by analysis with UV–Vis spectroscopic technique with a wavelength range of 200–800 nm. UV–Vis absorption spectra for zinc oxide nanoparticles synthesized from pomegranate leaf (ZnO-NPs-PL) showed an absorbance peak at 284 nm and another strong peak at 357 nm, while in the case of zinc oxide nanoparticles synthesized from flowers (ZnO-NPs-PF), an excitation absorption peak was observed at 345 nm (Figure 5). The shift in peak to a lower wavelength is caused by the presence of a blue shift which is observed as particle size reduces [43,44]. The absorption peaks obtained show the formation of zinc oxide nanoparticles following the green synthesis method, and also indicate combined vibration of electrons of the nanoparticles with the light waves. The peak values obtained are close to the characteristic line of the light absorption range of zinc oxide nanoparticles at 360–380 nm. A similar result was reported by Ezealisiji et al. [45], where a strong peak at 359 nm was obtained. In a study conducted by Tensingh and Lega [46], where nanorod-shaped zinc oxide nanoparticles with a particle size range of 20–100 nm were synthesized, a corresponding absorption peak at 354 nm was obtained. A study of the *Magnifera indica*-mediated synthesis of zinc oxide nanoparticles carried out by Rajeshkumar et al. [47] yielded an absorption peak of 355 nm.

### 3.3. Fourier Transform Infrared Spectroscopy (FTIR)

**Figure 6:**
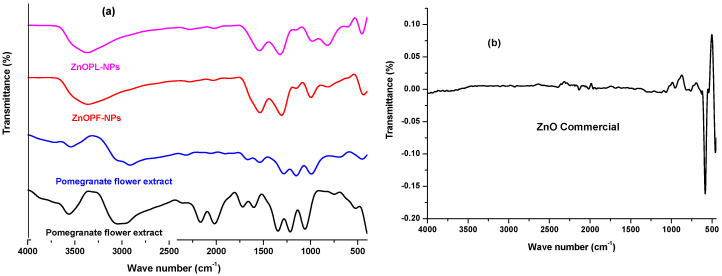
FTIR spectrum for (**a**) pomegranate leaf extract, pomegranate flower extract, pomegranate leaf extract-mediated zinc oxide nanoparticles (ZnO-NPs-PL), pomegranate flower extract-mediated zinc oxide nanoparticles (ZnO-NPs-PF) and (**b**) ZnO commercial sample.

FTIR analysis helps to identify the functional groups present in the compound on the surface on the nanoparticles, thereby providing molecular information of the molecules and biomolecules present in the plant extracts that participated in the synthesis of nanoparticles. FTIR analysis for this study was done in the spectral range of 4000 to 400 cm^−1^. The IR spectra for the plant extracts and obtained nanoparticles are shown in Figure 6a while the FTIR spectra of ZnO commercial sample is shown in Figure 6b. Strong peaks that show chemical structures for pomegranate leaf extract were observed at 3560 and 3700 cm^−1^, which fall within the range of 3000–3700 cm^−1^. This absorption range confirms the presence of the O-H stretch bond of free hydroxyl groups, which signifies that the pomegranate leaf contains flavonoids, polyphenols and alcohol functional groups that have different hydrogen bonds. Every bond type observed in FTIR analysis has a different frequency of vibration. Absorbance peaks were also obtained at 2936 cm^−1^, indicating the O-H stretch bond of carboxylic acid functional groups, and the -C-H stretch of alkanes. A less pronounced peak at 2100 cm^−1^ represents the -C≡C- stretch of alkynes and C≡N stretch of nitriles. Not so prominent peaks in the 1715–1727 cm^−1^ range confirm the presence of C=O carbonyl groups in the molecule, the C=O stretch of ketones, aldehydes, saturated aliphates and the stretch of α, β unsaturated esters. A less prominent absorption peak of 1588 cm^−1^ is indicative of C-C stretching (in ring) of aromatics and N-H bending of primary amines. Other absorption peaks obtained at 1352 cm^−1^ are representative of the C-H rock of alkanes and N=O symmetric stretching of nitro compounds. Peaks at 1215 cm^−1^ represent the C-O stretch of alcohols, carboxylic acids, esters and ethers. A 1053 cm^−1^ peak represents the C-N stretch of aliphatic amines. In addition, a less pronounced peak represents the N-H wag of primary and secondary amines, and C-H out of plane bend of aromatics indicated by 765 cm^−1^. A peak of 565 cm^−1^ shows the presence of the C-Br stretch of alkyl halides.

Bands for pomegranate flower were obtained at peaks 541, 752, 1053, 1215, 1339, 1613, 1727, 2026, 2175, 3060 and 3585 cm^−1^. The peak seen at 541 cm^−1^ represents a medium C-Br stretch of alkyl halides, 752 cm^−1^ denotes the C-Cl stretch of alkyl halides, C-O out of plane bending of aromatics, N-H wag of primary and secondary amines and a medium =C-H bend of alkenes. The peak at 1053 cm^−1^ represents the -C-O stretch of alcohols, carboxylic acid esters and ether functional groups. The 1215 cm^−1^ peak is representative of the C-N stretch of aliphatic amines and a medium C-O stretch of alcohol. The absorbance peak 1352 cm^−1^ indicates the N-O stretch of nitro compounds and a weak C-H rock of the alkane functional group. Peak 1613 cm^−1^ represents N-H bend of primary amines and peak 1727 cm^−1^ represents the C=O stretch of ketones, aldehydes, saturated aliphatics, αβ-unsaturated esters and the stretch of carbonyls. The band at 2175 cm^−1^ is due to a weak C≡C stretch of alkyne groups. The band seen at 3060 cm^−1^ indicates the O-H stretch of carboxylic acids. The band at 3585 cm^−1^ is due to the O-H stretch of H-bonded alcohols and the free hydroxyl O-H stretch vibration of phenol functional groups. On the other hand, the FTIR spectra shown in Figure 6b for the ZnO commercial sample showed peaks at 584 cm^−1^ which are characteristics of the Zn-O stretch. The absence of the O-H stretch band in the spectra for the commercial ZnO, which was pronounced in the spectra for ZnO-NPs-PF and ZnO-NPs-PL shown in Figure 6a further confirms that the extracts from the leaves and flowers of pomegranate did serve as reducing and capping agents for the synthesis of the ZnO-NPs used in this study.

FTIR spectra for zinc oxide nanoparticles prepared from pomegranate leaf and flower extracts are shown in Figure 6. ZnO-NPs-PL absorbed at peak values of 536, 690, 891, 1053, 1389, 1600, 2063, 2324 and 3394 cm^−1^, while for ZnO-NPs-PF, absorption peaks were seen at 516, 891, 1065, 1389, 1588, 2075, 2312 and 3398 cm^−1^. The bands observed at 536 cm^−1^ (ZnO-NPs-PL) and 516 cm^−1^ (ZnO-NPs-PF) correspond to the stretch band of zinc and oxygen (Zn-O), confirming synthesis of zinc oxide nanoparticles. Broad peaks absorbed at 3411 cm^−1^ and 3398 cm^−1^ for ZnO-NPs-PL and ZnO-NPs-PF, respectively, can be attributed to the asymmetric and symmetric stretching of the H-O-H vibration mode of phenol [48]. Broad bands at 1600 cm^−1^ (ZnO-NPs-PL) and 1588 cm^−1^ (ZnO-NPs-PF) may correspond to a medium C=C ring stretch absorption of aromatics and N-H bend of primary amines, where a shift in band to a lower frequency is observed. A not so pronounced peak of 1053 cm^−1^ (ZnO-NPs-L) and a pronounced peak of 1065 cm^−1^ (ZnO-NPs-F) may indicate the C-O stretch of alcohols, carboxylic acids, esters, ethers and C-N stretching of aliphatic amines. The bands at 891 cm^−1^ represent the C-H out of plane bending of aromatics and N-H wag of primary and secondary amines, while the peak of 690 cm^−1^ observed in ZnO-NPs-PL only indicates the presence of the C-Br stretch of alkyl halides. Shifts in the vibration band of the carboxylic groups (from 2936 cm^−1^ of PL extract and 3060 cm^−1^ of PF extract to a lower wavelength of 2655 cm^−1^ (ZnO-NPs-PL) and 2658 cm^−1^ (ZnO-NPs-PF)), and phenol groups (from 3560–3700 cm^−1^ range for PL extract and 3585 cm^−1^ PF extract to a lower wavelength of 3411 cm^−1^ (ZnO-NPs-L) and 3398 cm^−1^ (ZnO-NPs-F)), coupled with the absence of ketone in the ZnO-NPs-PL and ZnO-NPs-PF spectra, signify that there was an interaction between the plant leaf and flower extracts and the metal salts. The absence of ketones in the spectrum for synthesized ZnO-NPs-PL and ZnO-NPs-PF may probably mean that the ketone was used up during the synthesis of zinc oxide nanoparticles, where it acted as a reducing and capping agent. Carboxylic and alcohol compounds can bind metals and may form metal nanoparticles by stabilizing the medium and preventing agglomeration [40]. The presence of functional groups within the leaf and flower extract of pomegranate plants, such as amines, alcohols, ketones, phenols, carboxylic acids and alkenes, may have contributed to the reduction of Zn^2+^. In addition, bio-capping during synthesis can be attributed to the action of carboxylic and phenolic acid, while the stability of synthesized zinc oxide nanoparticles is linked to the presence of free amino and carboxylic group interactions with the zinc oxide nanoparticle surface.

### 3.4. X-ray Diffraction (XRD) Analysis

**Figure 7:**
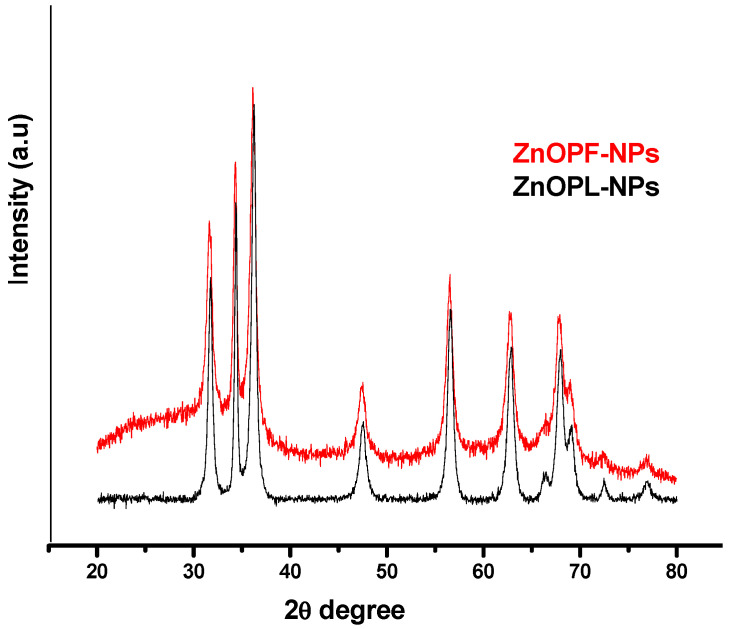
XRD spectra of pomegranate leaf extract-mediated zinc oxide nanoparticles (ZnO-NPs-PL) and pomegranate flower extract-mediated zinc oxide nanoparticles (ZnO-NPs-PF).

Characterization of biosynthesized zinc oxide nanoparticles via pomegranate leaf and flower extracts was analyzed by X-ray diffraction. This was done to ascertain the purity and crystalline structure of the metal oxide nanoparticles [49,50]. The obtained diffraction peaks at 2θ values, as shown in Figure 7, were 31.71°, 34.34°, 36.25°, 47.54°, 56.62°, 62.76°, 68.00°, 69.10°, 72.43° and 72.43° for zinc oxide nanoparticles synthesized via pomegranate leaf extract, and 31.74°, 34.42°, 36.13°, 47.54°, 56.53°, 62.84°, 67.93°, 69.05° and 72.69° for the pomegranate flower extract-mediated zinc oxide nanoparticles. These peak values relate to the crystal or lattice plane of (100), (002), (101), (102), (110), (103), (200), (112), (201) and (202), according to the Joint Committee on Powder Diffraction Studies Standards (JCPDS card number 008, 82–1042 and 5–0664). The correspondent plane, also known as Bragg’s reflection line, suggests that the synthesized metal oxide nanoparticles might be of spherical crystalline structure. Similar results were obtained by Fu and Fu. [15], who biosynthesized zinc oxide nanoparticles via *Plectranthus amboinicus* leaf extract and reported diffraction peaks at 31.899°, 34.420°, 36.145°, 47.987°, 56.502°, 63.101°, 67.958° and 69.014° that correspond to (100), (002), (101), (102), (110), (103), (112) and (201). The XRD spectra of pomegranate leaf- and flower-mediated zinc oxide nanoparticles obtained from the study showed no other external peaks, indicating purity of the synthesized nanoparticles and suggesting the green method of synthesis used can be deployed to obtain nanoparticles of high purity [48]. From the figures, the XRD pattern shows both strong broad and narrow diffraction peaks, which signify that the biosynthesized zinc oxide nanoparticles are crystalline in nature [39]. However, the broad peak is an indication that the nanoparticles are small and fine particles (nanoscale crystalline particles) while the narrow or low-intensity peak is an indication of low crystallinity of the nanoparticles [51]. From the XRD spectral pattern, the mean particle diameter of the biosynthesized zinc oxide nanoparticles can be calculated following the line width of the maximum intensity reflection peak using the Debye–Scherrer equation [47,52]:

(1) $$d=\frac{k\lambda }{\beta Cos\theta }$$

where *d* denotes the mean particle size

*k* = 0.89 (shape factor)
*λ* = X-ray wavelength (1.5406 A°)
*β* denotes full width at half maximum (FWHM) in radians
*Ɵ* denotes the Bragg diffraction angle.

From the above equation, the crystalline particle size derived for pomegranate leaf-mediated zinc oxide nanoparticles and pomegranate flower-mediated zinc oxide nanoparticles was 57.75 and 52.50 nm, respectively. These are close to the particle size values reported by Yuvakkumar et al. [7] and Dhanemozhi et al. [53], who obtained “D” values of 50.95 and 54.84 nm, respectively.

### 3.5. Scanning Electron Microscopy with Energy Dispersive X-ray (EDX)

**Figure 8:**
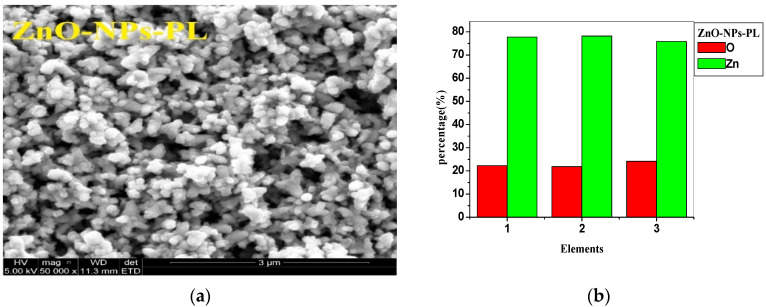
(**a**) SEM image of pomegranate leaf-mediated zinc oxide nanoparticles. (**b**) EDX spectrum of pomegranate leaf-mediated zinc oxide nanoparticles.

**Figure 9:**
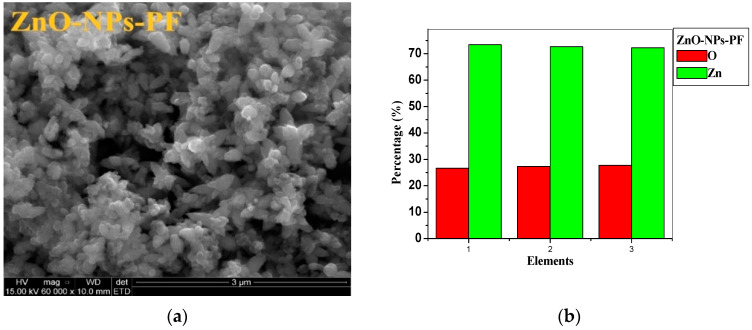
(**a**) SEM image of pomegranate flower-mediated zinc oxide nanoparticles. (**b**) EDX spectrum of pomegranate flower-mediated zinc oxide nanoparticles.

SEM imaging with EDX analysis was done to further confirm the presence of synthesized nanoparticles. From the SEM images of Figure 8a and Figure 9a, the zinc oxide nanoparticles synthesized from pomegranate leaf (ZnO-NPs-PL) shows an uneven round structure, which is similar to that reported by Umar et al. [37] and Wali et al. [54], and the presence of agglomerated nanoparticles can be seen on the SEM image. The SEM image of ZnO-NPs-PF (Figure 9a) also reveals an irregularly shaped microstructure with protrusions which seem flower-like. EDX analysis to confirm elemental compositions of the synthesized nanoparticles reveals the presence of zinc and oxygen components. The elemental analysis of the nanoparticles shows 77.24% zinc and 22.74% oxygen elements for ZnO-NPs-PL (Figure 8b), while ZnO-NPs-PF (Figure 9b) shows a 72.75% zinc and 27.25% oxygen elemental composition. This indicates that the synthesized nanoparticles are in their purest form.

### 3.6. Antibacterial Activity

**Figure 10:**
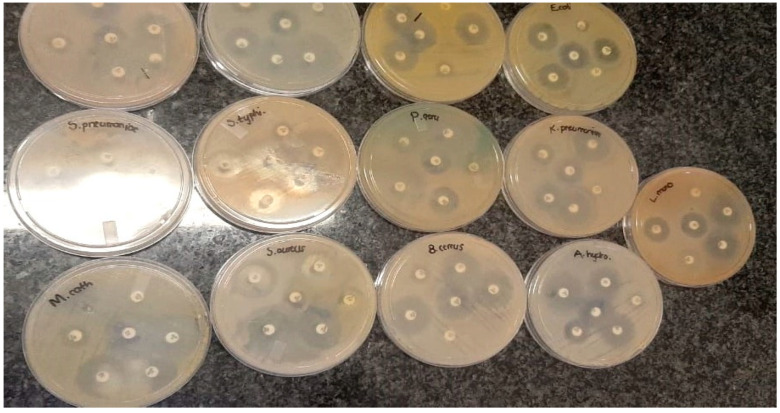
Antibiogram tests showing zones of inhibition against pathogenic strains.

Antibacterial resistance remains a major bottleneck to the treatment of infectious disease in healthcare systems, animal husbandry and in the food production sector. The antibiogram test against the pathogenic strains (Table 1) used in the study was evaluated by the disc diffusion method, following the guidelines of the Clinical Laboratory Standard Institute (CSLI). The results were represented by measuring the diameter of zones of inhibition in mm. Table 2 gives an overview of the report of the antibiogram test, and Figure 10 is a pictorial representation of the conducted antibiogram test. From the results represented in Table 2, *S. aureus* ATCC 25923, *E. coli* ATCC 25922, *S. pneumoniae* ATCC 27336, *B. cereus* ATCC 10876, *M. catarrhalis* ATCC 25240, *A. hydrophila* ATCC 7966, *S. diarizonae* ATCC 12325, *E. faecalis* ATCC 29212, *E. faecium* ATCC 6569 and *K. pneumoniae* ATCC 13883 exhibited susceptibility to tetracycline, while *L. monocytogenes* ATCC 19115 showed resistance to tetracycline antibiotics.

*S. aureus* ATCC 25923, *E. coli* ATCC 25922, *A. hydrophila* ATCC 7966, *E. faecalis* ATCC 29212, *K. pneumoniae* ATCC 13883 and *M. catarrhalis* ATCC 25240 were resistant to ampicillin, but *B. cereus* ATCC 10876, *E. faecium* ATCC 6569, *S. diarizonae* ATCC 12325, *S. pneumoniae* ATCC 27336 and *L. monocytogenes* ATCC 19115 were susceptible to ampicillin. All thirteen pathogenic strains used in the study showed susceptibility to amikacin, Augmentin and gentamicin. With the exception of *E. faecium*, all other pathogenic strains in the study were susceptible to ciprofloxacin and norfloxacin. *S. aureus* ATCC 25923, *E. coli* ATCC 25922, *A. hydrophila* ATCC 7966, *S. diarizonae* ATCC 12325, *B. cereus* ATCC 10876, *E. faecium* ATCC 6569, *K. pneumoniae* ATCC 13883, *M. catarrhalis* ATCC 25240, *S. pneumoniae* ATCC 27336 and *L. monocytogenes* ATCC 19115 were resistant to cefotaxime, however, *S. typhi*, *E. faecalis* ATCC 29212 and *Pseudomonas aeruginosa* ATCC 27853 exhibited susceptibility to the cefotaxime antibiotic.

Generally, cefalexin (CFX) and ampicillin were the most resisted antibiotics, while amikacin, Augmentin and gentamicin were the most effective antibiotics (see Table 2 for zones of inhibition measurement in mm).

### 3.7. Antibacterial Activity of Synthesized Metal Oxides Nanoparticles

**Figure 11:**
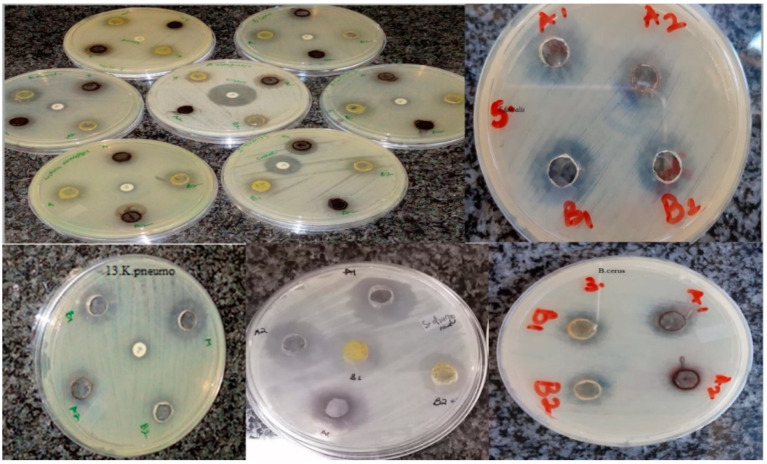
Plates showing the zones of inhibition obtained upon testing biosynthesized nanoparticles against bacterial strains.

The antibacterial efficacy of the synthesized zinc oxide nanoparticles was evaluated against 13 pathogenic strains using the agar well diffusion method. Different concentrations (50 µg/mL, 500 µg/mL, 1000 µg/mL and 5000 µg/mL) of the nanoparticles were prepared and used. Synthesized zinc oxide nanoparticles mediated via pomegranate leaf extract in this study are represented as ZnO-NPs-PL while zinc oxide nanoparticles mediated via pomegranate flower extract are represented as ZnO-NPs-PF. Antimicrobial activity exhibited by the synthesized nanoparticles, which prevents the growth of bacteria, can be seen in the form of a clear zone of inhibition, as seen in Figure 11. From Table 3, using the 5000 µg/mL concentration as a standard, the synthesized zinc oxide nanoparticles exhibited activity against all bacterial strains used in the study. *E. faecium* ATCC 6569 and *S. diarizonae* ATCC 12325 showed more susceptibility, with high zones of inhibition (mean value) of 19.33 ± 1.15 mm and 19.00 ± 1.00 mm, respectively, for ZnO-NPs-PL. With respect to 5000 µg/mL ZnO-NPs-PF, *E. faecium*, *S. pneumoniae* and *S. diarizonae* exhibited high susceptibility, with zones of inhibition at 21.50 ± 0.50 mm, 19.00 ± 0.50 mm and 17.50 ± 0.50 mm, respectively. The ability of zinc oxide nanoparticles to exert antimicrobial activity has been documented. In a study by Umar et al. [37], the antimicrobial activity of zinc oxide nanoparticles synthesized via *A. lebbeck* stem extract was evaluated against *B. cereus*, *S. aureus*, *E. coli*, *K. pneumoniae* and *S. typhi.* Khatami et al. [49] documented the antiparasitic and antimicrobial effect of stevia-mediated, rectangular-shaped zinc oxide nanoparticles against *Leishmania major*, *S. aureus* and *E. coli* and showed that an increase in the concentration of zinc oxide nanoparticles elicited maximum cytotoxic effect. Ibrahem et al. [55] also recounted the concentration-dependent manner of zinc oxide nanoparticles. Nazoori and Kariminik [56] investigated the antimicrobial efficiency of zinc oxide nanoparticles against ten (10) human pathogenic organisms, and reported that the minimum concentration of zinc oxide that can inhibit or prevent the growth of most of *S. aureus*, *S. marcescens* and *E. coli* cultures was 2.5mg/mL of synthesized ZnO-NPs, while the minimum concentration required to kill off the bacterial strains was 10mg/mL. In another study by Akbar et al. [33], ZnO nanoparticles were synthesized and their antimicrobial potential against foodborne pathogens *S. typhimurium* and *S. aureus* was evaluated. The mode of antimicrobial action by nanoparticles is still not clear, but studies have suggested that zinc oxide nanoparticles exert antibacterial effects on microorganisms by ROS production [57], membrane leakage of intracellular content, DNA and protein damage and toxic ion release [18]. Zinc oxide is generally regarded as safe by the Food and Drug Administration (FDA). Zinc oxide nanoparticles have different applications, such as in food science where they serve as preservatives, in pharmaceuticals as drug delivery agents and in industrial applications like textiles.

**Figure 12:**
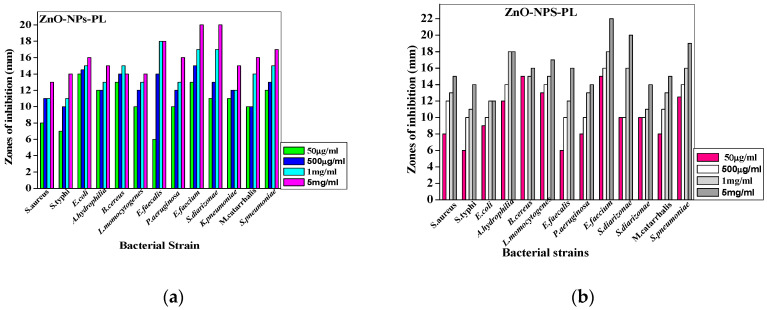
(**a**) Bar graphs showing the effect of different concentrations (50 µg/mL, 500 µg/mL, 1 mg/mL and 5 mg/mL) on antimicrobial activity of ZnO-NPs-PL against various microorganisms. (**b**) Bar graphs showing the effect of different concentrations (50 µg/mL, 500 µg/mL, 1 mg/mL and 5 mg/mL) on antimicrobial activity of ZnO-NPs-PF against various microorganisms.

The ability of the prepared zinc oxide nanoparticles to show activity against the tested pathogenic stains could be attributed to oxidative stress caused by ROS generation, which leads to protein and bacterial DNA damage [57]. In the study, the prepared metal oxide nanoparticles could have produced ROS that elicited inhibition against the bacterial strains. In a study by Padmavathy and Vijayaraghavan, [58], a high generation of ROS was cited as the main cause of bacterial cell death upon treatment with synthesized zinc oxide nanoparticles. Additionally, in another study by Raghupathi et al. [13], the generation of ROS-like hydrogen peroxide, hydroxyl radicals and superoxide anions in the presence of UV light, which caused cell toxicity, was reported as the mechanism of antibacterial inhibition. The antibacterial effect of zinc oxide nanoparticles on multidrug-resistant ESKAPE pathogen *Acinetobacter baumannii* was reported by Tiwari et al. [57], who recounted that ROS generation, which caused oxidative degradation of lipids, leakage of membrane reducing sugar, proteins and DNA and eventual loss of cell viability, was the mode of action for the zinc oxide nanoparticles. From Table 3 and Table 4 above, and also Figure 12a,b below, it is evident that enhanced antibacterial activity of metal oxide nanoparticles increases with concentration. With an increase in nanoparticle concentration, bacterial growth generally reduces. A similar report was given by Liu et al. [59], who demonstrated that an increase in the concentration of the prepared nanoparticles increased the antimicrobial activity of zinc oxide nanoparticles against *E. coli*. Pati et al. [16] also reported on the dose-dependent activity of nanoparticles against bacterial strains.

### 3.8. Determination of Minimum Inhibitory Concentrations (MICs) of the Synthesized Metal Oxide Nanoparticles

The antibacterial effects of the prepared zinc oxide nanoparticles mediated via pomegranate leaf and flower extracts were also evaluated using the minimum inhibitory concentration technique. This was done to ascertain the lowest concentration of the prepared metal oxide nanoparticles that can inhibit or stop bacterial growth. From the tests carried out on the 96-well titer plate, the lowest concentration of ZnO-NPs-PF that could completely inhibit growth of *S. aureus*, *E. faecalis*, *E. coli*, *E. faecium*, *L. monocytogenes*, *K. pneumoniae*, *S. pneumoniae* and *S. typhi* was at 1250 µg/mL, while for *B. cereus* and *M. catarrhalis*, the minimum inhibitory concentration was achieved at 0.61 ± 0.54 µg/mL. MIC values for *P. aeruginosa* and *A. hydrophila* were 0.83 ± 0.36 µg/mL and 0.82 ± 0.36 µg/mL, respectively. For ZnO-NPs-PL, the minimum inhibitory concentrations against *B. cereus*, *P. aeruginosa*, *E. faecalis*, *A. hydrophila*, *S. pneumoniae*, *E. faecium*, *S. aureus*. *E. coli*, *L. monocytogenes*, *M. catarrhalis*, *S. typhi* and *K. pneumoniae* were 0.93 ± 0.54 µg/mL, 0.88 ± 0.6 µg/mL, 1.25 mg/mL, 0.85 ± 0.6 µg/mL, 0.93 ± 0.54 µg/mL, 0.816 ± 0.375 µg/mL, 1250 µg/mL, 1250 µg/mL, 0.71 ± 0.48 µg/mL, 1250 µg/mL, 0.6 µg/mL and 1250 µg/mL, respectively.

### 3.9. Killing Time

**Figure 13:**
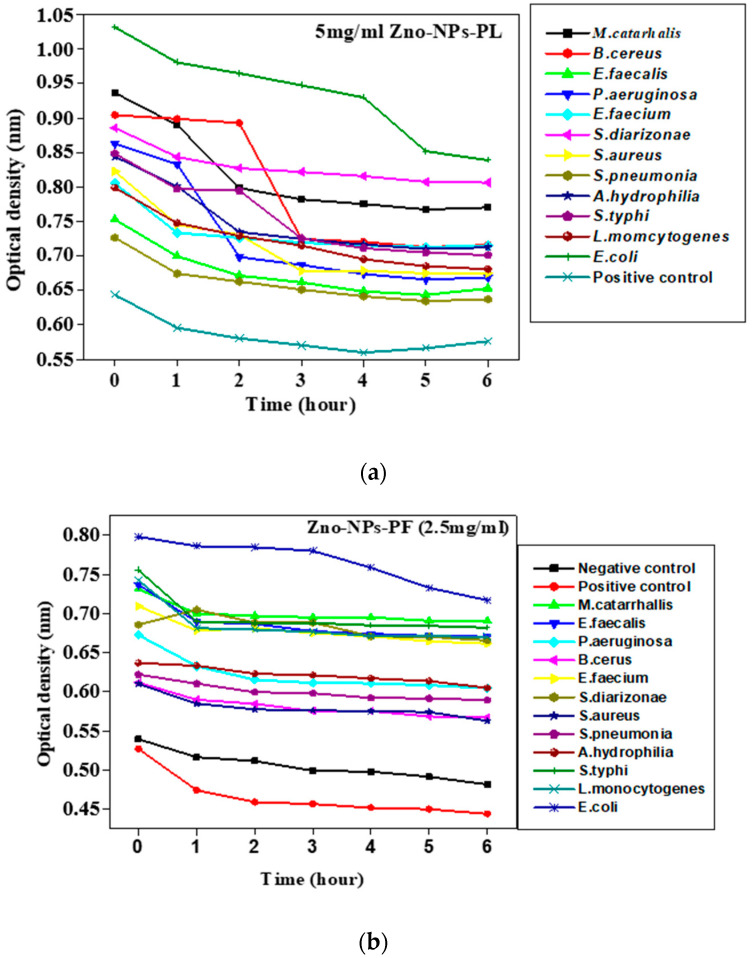

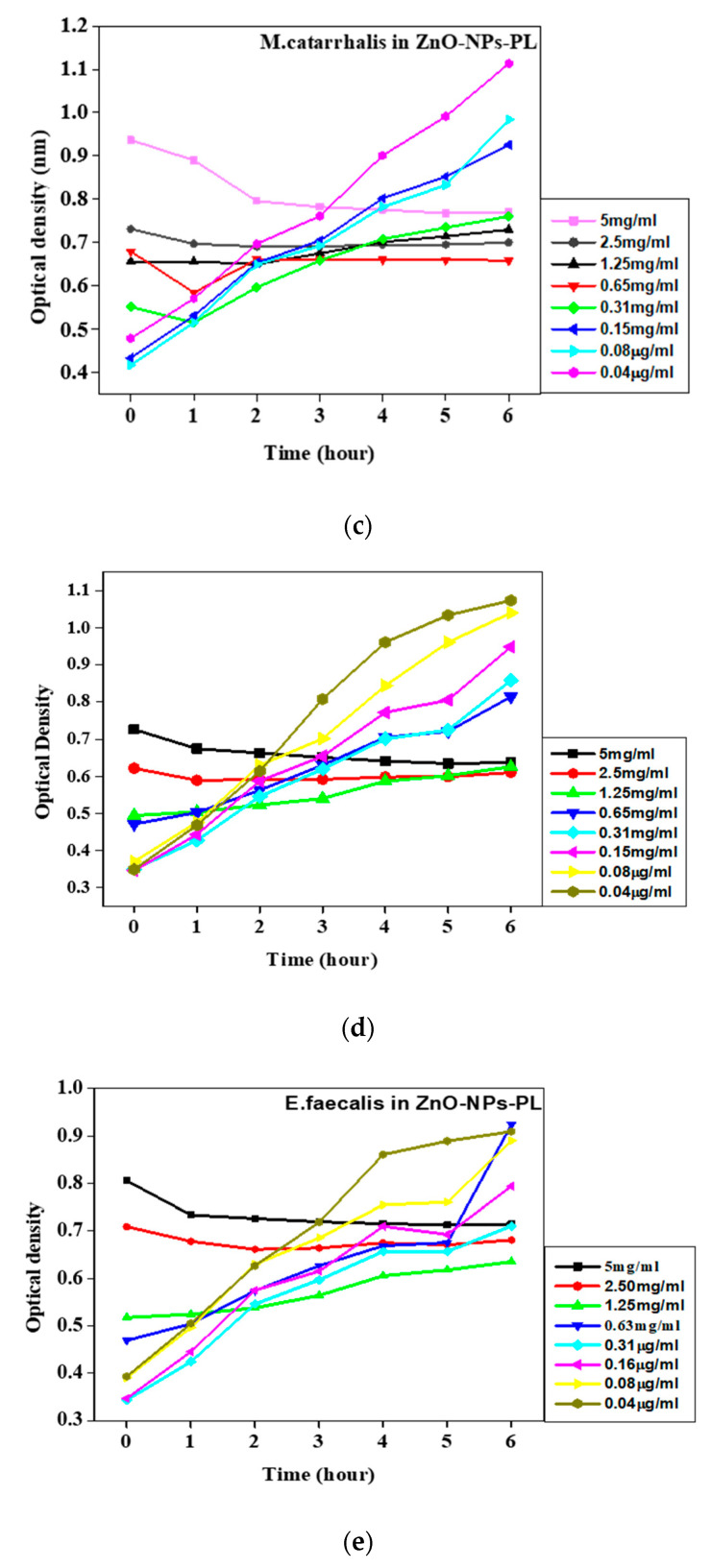
(**a**) Effect of time on persistence (survivability) of individual pathogenic strains in 5 mg/mL zinc oxide nanoparticles. (**b**) Effect of time on persistence (survivability) of individual pathogenic strains in 2.5 mg/mL zinc oxide nanoparticles. (**c**) Effect of time on persistence (survivability) of *M. catarrhalis* in zinc oxide nanoparticles. (**d**) Effect of time on persistence (survivability) of *S. aureus* in zinc oxide nanoparticles. (**e**) Effect of time on persistence (survivability) of *E. faecalis* in zinc oxide nanoparticles.

The survivability of the different pathogenic strains was evaluated in different concentrations of synthesized metal oxide nanoparticles. Growth analysis was measured and recorded by monitoring the optical density at 630 nm over time. From the graph shown in Figure 13a at 5000 µg/mL of zinc oxide nanoparticles, it can be seen that the growth of the pathogenic strains *Staphylococcus aureus* ATCC 25923, *E. coli* ATCC 25922, *S. pneumoniae* ATCC 27336, *P. aeruginosa* ATCC 27853, *B. cereus* ATCC 10876, *M. catarrhalis* ATCC 25240, *A. hydrophila* ATCC 7966, *S. diarizonae* ATCC 12325, *E. faecalis* ATCC 29212, *E. faecium* ATCC 6569, *S. typhi*, and *L. monocytogenes* ATCC 19115 was totally inhibited, although *B. cereus* and the *P. aeruginosa* strains exhibited growth, or were in the lag phase, where the bacteria were adjusting to the media they were placed in, till the second hour, where a very sharp decline in growth profile was observed. At 2.5 mg (Figure 13b), effective inhibition was also observed. In the case of the *E. coli* strain, cellular activity occurred from 0 h to the first (1) hour, after which the exponential phase occurred from hour two until hour four, with zinc oxide nanoparticles disrupting the metabolic activity, and by the fifth hour there was a stationary phase, which subsequently led to the death phase at the sixth hour. Figure 13c–e show the effect of time on survivability in different concentrations of zinc oxide nanoparticles for *M. catarrhalis*, representing Gram-negative organisms, and *S. aureus* for Gram-positive organisms.

## 4. Conclusions

The effects of different concentrations of zinc oxide nanoparticles against pathogenic bacterial strains were evaluated. High concentrations of nanoparticles at 5 mg/mL effectively inhibited the growth of bacteria. From the zones of inhibition obtained, the zinc oxide produced from leaf and flower extracts showed consistency, inhibiting all bacterial strains used in the analysis. The mechanism of action of inhibiting bacterial growth is suggested to be by production of ROS upon attachment to the bacterial cell membrane, thereby causing damage to the cell membrane and protein dysfunction. Liu et al. [59] report that upon treatment of *E. coli* with zinc oxide nanoparticles, the particles fixed onto the cell membrane of *E. coli*, causing deformity of the membrane and disorganization of the intracellular structures. Kadiyala et al. [20] investigated the antibacterial effect of zinc oxide nanoparticles on multidrug-resistant *S. aureus* and indicated that the generation of ROS alone cannot be the main antibacterial mode of action. An increase in the concentration of nanoparticles yielded a large zone of inhibition, signifying a greater antibacterial effect. From the analysis carried out, the minimum concentration of zinc oxide that could effectively inhibit the growth of microorganisms used in the study ranged from 0.6 µg/mL to 2500 µg/mL, depending on the bacterial strain. From these findings, we recommended that further cytotoxicity studies be carried out to determine the safety properties of these nanoparticles against representatives of host cells such as erythrocytes or fibroblasts.

## Figures and Tables

**Table 1:** List of bacterial strains.

S/N	Bacteria	Strain Number	Gram Stain
1	*Staphylococcus aureus*	ATCC 25923	Gram-positive
2	*Streptococcus pneumoniae*	ATCC 27336	Gram-positive
3	*Bacillus cereus*	ATCC 10876	Gram-positive
4	*Enterococcus faecalis*	ATCC 29212	Gram-positive
5	*Listeria monocytogenes*	ATCC 19115	Gram-positive
6	*Enterococcus faecium*	ATCC 6569	Gram-positive
7	*Klebsiella pneumoniae*	ATCC 13883	Gram-negative
8	*Salmonella typhi*	Lab isolate	Gram-negative
9	*Escherichia coli*	ATCC 25922	Gram-negative
10	*Pseudomonas aeruginosa*	ATCC 27853	Gram-negative
11	*Moraxella catarrhalis*	ATCC 25240	Gram-negative
12	*Aeromonas hydrophila*	ATCC 7966	Gram-negative
13	*Salmonella diarizonae*	ATCC 12325	Gram-negative

**Table 2:** Antibiogram resistance profiles of the isolates.

S/N	Bacterial Isolates	TET	AMP	AK	GEN	AUG	CIP	NOR	CFX
1	*Staphylococcus aureus*	S	R	S	S	S	S	S	R
2	*Salmonella typhi*	NE	NE	S	S	S	S	S	S
3	*Escherichia coli*	S	R	S	S	S	S	S	R
4	*Aeromonas hydrophila*	S	R	S	S	S	S	S	R
5	*Enterococcus faecalis*	S	R	S	S	S	S	S	S
6	*Pseudomonas aeruginosa*	NE	NE	S	S	S	S	S	S
7	*Bacillus cereus*	S	S	S	S	S	S	S	R
8	*Enterococcus faecium*	S	S	S	S	S	R	R	R
9	*Salmonella diarizonae*	S	S	S	S	S	S	S	R
10	*Klebsiella pneumoniae*	S	R	S	S	S	S	S	R
11	*Moraxella catarrhalis*	S	R	S	S	S	S	S	R
12	*Streptococcus pneumoniae*	S	S	S	S	S	S	S	R
13	*Listeria monocytogenes*	R	S	S	S	S	S	S	R

Key: S indicates susceptibility, R indicates resistance. NE: indicates not evaluated. TET (tetracycline), AMP (ampicillin), AK (amikacin), GEN (gentamicin), AUG (Augmentin), CIP (ciprofloxacin), NOR (norfloxacin), CFX (cefotaxime).

**Table 3:** Mean zones of inhibition value (mm) produced by zinc oxide nanoparticles mediated via pomegranate (*Punica granatum*) leaf extract.

S/N	Bacterial Isolates	50 µg/mL(mm)	100 µg/mL(mm)	1000 µg/mL(mm)	5000 µg/mL(mm)
1	*Staphylococcus aureus*	7.00 ± 1.00 ^a^	10.50 ± 0.87 ^a^	11.33 ± 0.58 ^a^	13.00 ^a^
2	*Salmonella typhi*	6.33 ± 0.58 ^a^	10.00 ^a^	11.17 ± 0.29 ^a^	14.33 ± 0.58 ^a^
3	*Escherichia coli*	13.33 ± 1.15 ^d^	13.83 ± 0.76 ^c^	15.00 ± 1.00 ^b^	15.67 ± 1.53 ^a^
4	*Aeromonas hydrophila*	11.00 ± 2.65 ^b^	12.33 ± 0.58 ^a^	13.33 ± 0.58 ^a^	14.67 ± 1.15 ^a^
5	*Bacillus cereus*	10.33 ± 2.52 ^b^	13.67 ± 0.58 ^c^	15.33 ± 0.58 ^b^	14.33 ± 1.5 ^a^
6	*Listeria monocytogenes*	10.00 ± 1.00 ^b^	12.00 ± 1.00 ^a^	12.33 ± 1.15 ^a^	13.67 ± 1.53 ^a^
7	*Enterococcus faecalis*	6.33 ± 0.29 ^a^	14.33 ± 0.58 ^d^	17.33 ± 1.15 ^d^	18.00 ^b^
8	*Pseudomonas aeruginosa*	9.67 ± 1.53 ^b^	11.33 ± 0.58 ^a^	12.67 ± 1.53 ^a^	15.33 ± 1.53 ^a^
9	*Enterococcus faecium*	12.67 ± 0.58 ^c^	15.17 ± 0.29 ^e^	16.67 ± 1.53 ^c^	19.33 ± 1.15 ^c^
10	*Salmonella diarizonae*	10.83 ± 1.26 ^b^	12.00 ± 1.73 ^a^	15.33 ± 1.15 ^b^	19.00 ± 1.00 ^c^
11	*Klebsiella pneumoniae*	9.67 ± 1.53 ^b^	11.67 ± 1.53 ^a^	12.00 ± 1.00 ^a^	14.00 ± 2.65 ^a^
12	*Moraxella catarrhalis*	8.67 ± 1.15 ^b^	9.67 ± 1.53 ^a^	13.33 ± 2.08 ^a^	14.00 ± 2.65 ^a^
13	*Streptococcus pneumoniae*	10.67 ± 2.31 ^b^	12.67 ± 0.58 ^b^	14.67 ± 1.53 ^a^	16.67 ± 0.58 ^a^

Superscripts ^a, b, c, d, e^ indicate the mean ± standard deviation of the bacteria growth inhibition zone diameter data for the produced by zinc oxide nanoparticles from pomegranate (*Punica granatum*) leaf extract on the isolates. In each concentration column, all values with the same alphabetic superscript are not significantly different.

**Table 4:** Mean zones of inhibition value (mm) produced by zinc oxide nanoparticles mediated via pomegranate (*Punica granatum*) flower extract.

S/N	Bacterial Isolates	50 µg/mL(mm)	100 µg/mL(mm)	1000 µg/mL(mm)	5000 µg/mL(mm)
1	*Staphylococcus aureus*	7.00 ± 1.00 ^a^	12.33 ± 0.58 ^c^	12.50 ± 0.50 ^b^	15.00 ± 1.00 ^a^
2	*Salmonella typhi*	5.67 ± 0.58 ^a^	8.67 ± 1.15 ^a^	12.00 ± 1.00 ^a^	14.53 ± 0.23 ^a^
3	*Escherichia coli*	8.67 ± 0.58 ^c^	10.00 ± 1.00 ^a^	14.00 ± 1.00 ^c^	14.50 ± 0.50 ^a^
4	*Aeromonas hydrophila*	10.33 ± 2.05 ^e^	13.83 ± 0.29 ^d^	17.33 ± 1.15 ^g^	17.50 ± 0.50 ^e^
5	*Bacillus cereus*	10.67 ± 2.08 ^e^	12.33 ± 0.58 ^c^	14.50 ± 1.32 ^d^	16.33 ± 0.58 ^c^
6	*Listeria monocytogenes*	6.33 ± 0.58 ^a^	14.33 ± 0.58 ^d^	14.67 ± 0.58 ^e^	16.50 ± 0.50 ^d^
7	*Enterococcus faecalis*	7.33 ± 1.15 ^a^	9.67 ± 0.58 ^a^	12.00 ± 1.00 ^a^	16.17 ± 0.15 ^c^
8	*Pseudomonas aeruginosa*	12.67 ± 0.58 ^g^	10.00 ± 1.00 ^a^	13.00 ^b^	14.00 ^a^
9	*Enterococcus faecium*	9.67 ± 0.58 ^d^	15.83 ± 0.76 ^e^	18.00 ^h^	21.50 ± 0.50 ^g^
10	*Salmonella diarizonae*	10.67 ± 0.58 ^e^	10.00 ^a^	16.00 ± 1.00 ^f^	18.50 ± 0.50 ^e^
11	*Klebsiella pneumoniae*	8.00 ± 1.00 ^b^	11.00 ± 1.00 ^b^	10.67 ± 1.15 ^a^	14.00 ^a^
12	*Moraxella catarrhalis*	12.00 ± 0.50 ^f^	12.00 ± 1.00 ^c^	11.67 ± 1.15 ^a^	15.33 ± 1.44 ^b^
13	*Streptococcus pneumoniae*	10.67 ± 2.30 ^e^	14.00 ± 1.00 ^d^	16.00 ^f^	19.00 ± 0.50 ^f^

Superscripts ^a, b, c, d, e, f, g, h^ indicate the mean ± standard deviation of the bacteria growth inhibition zone diameter data for the produced by zinc oxide nanoparticles from pomegranate (*Punica granatum*) flower extract on the isolates. In each concentration column, all values with the same alphabetic superscript are not significantly different.
